# Facile Functionalization of Poly(Dimethylsiloxane) Elastomer by Varying Content of Hydridosilyl Groups in a Crosslinker

**DOI:** 10.3390/polym11111842

**Published:** 2019-11-08

**Authors:** Seung Koo Park, Bong Je Park, Mee Jeong Choi, Dong Wook Kim, Jae Woong Yoon, Eun Jin Shin, Sungryul Yun, Suntak Park

**Affiliations:** 1 Human Enhancement & Assistive Technology Research Section, Artificial Intelligence Research Laboratory, Electronics and Telecommunications Research Institute, 218 Gajeong-ro, Yuseong-gu, Daejeon 34129, Korea; bjpark@etri.re.kr (B.J.P.); jjeong0527@etri.re.kr (M.J.C.); yjw60212@etri.re.kr (J.W.Y.); shin015511@etri.re.kr (E.J.S.); sungryul@etri.re.kr (S.Y.); 2 Advanced Materials Division, Korea Research Institute of Chemical Technology, 141 Gajeong-ro, Yuseong-gu, Daejeon 34114, Korea; dongwook@krict.re.kr

**Keywords:** poly(dimethylsiloxane), hydrosilylation, functionalization, permittivity, transparency, elasticity

## Abstract

Crosslinked poly(dimethylsiloxane) (PDMS) has been widely used as a dielectric elastomer for electrically driven actuators because it exhibits high elasticity, low initial modulus, and excellent moldability in spite of low dielectric constant. However, further improvement in the characteristics of the PDMS elastomer is not easy due to its chemical non-reactivity. Here, we report a simple method for functionalizing the elastomer by varying content of hydridosilyl groups in PDMS acted as a crosslinker. We synthesized poly(dimethylsiloxane-*co*-methylvinylsiloxane) (VPDMS) and poly(dimethylsiloxane-*co*-methylsiloxane) (HPDMS). Tri(ethylene glycol) divinyl ether (TEGDE) as a polar molecule was added to the mixture of VPDMS and HPDMS. TEGDE was reacted to the hydridosilyl group in HPDMS during crosslinking between VPDMS and HPDMS in the presence of platinum as a catalyst. Permittivity of the crosslinked film increased from ca. 25 to 36 pF/m at 10 kHz without a decline in other physical properties such as transparency and elasticity (*T* > 85%, *E* ~150 kPa, ɛ ~270%). It depends on the hydridosilyl group content of HPDMS. The chemical introduction of a new molecule into the hydridosilyl group in HPDMS during crosslinking would provide a facile, effective method of modifying the PDMS elastomers.

## 1. Introduction

Crosslinked poly(dimethylsiloxane) (PDMS) has been well known for an elastomer material for electrical-driven actuators, wearable devices, and artificial muscles because it can produce large and dynamically reversible deformation in response to an electric potential [1,2,3,4,5]. The crosslinked PDMS film is generally prepared from a reaction between vinyl or allyl and hydridosilyl groups in a liquid PDMS mixture via platinum (Pt)-catalyzed hydrosilylation [6,7,8]. In the mixture, PDMS with the hydridosilyl group is a so-called crosslinker. The crosslinked film has been extensively studied for dielectric elastomer actuators, despite low permittivity, because the viscous liquid PDMS mixture is moldable before crosslinking and the crosslinked film is exceedingly soft, stretchable, transparent, and high voltage-endurable.

High driving voltage over several kV is required for actuation of the PDMS elastomer, which limits electrical safety and miniaturization of the dielectric elastomer actuator integrated into operation system [3,4,5]. Dielectric property of the elastomer is crucial for reducing the driving voltage. However, the permittivity of the elastomer cannot be readily improved due to its chemical non-reactivity. Similarly, since the modification of PDMS is difficult, even though the liquid PDMS is one of promising materials for microfluidic devices, the other materials such as Teflon, glass, and parylene have been mainly studied for that purpose [9,10,11]. In order to improve the dielectric property of the film, organic polar groups such as halogen, sulfur, and cyano groups have been chemically introduced into the silicone polymer main chain in the film [12,13,14,15,16]. In this case, an additional synthetic process is required for preparation of the PDMS monomers and prepolymers to which the polar groups chemically attach. In simple ways, inorganic fillers with high dielectric constant such as titanium dioxide, zirconium dioxide, barium titanate, carbon, and silver nanoparticles are physically blended with PDMS [16,17,18,19,20,21,22]. However, the crosslinked PDMS film dispersed with the inorganic particle has shown significant increase of stiffness and opacity.

It has been reported that an azobenzene chromophore with hydroxyl groups was chemically attached to tetraethylorthosilicate (TEOS) during crosslinking reaction of hydroxy-terminated PDMS with TEOS in order to increase dielectric constant of the crosslinked PDMS film [23]. This method must be an efficient and convenient way of chemically introducing polar groups to a crosslinked PDMS. Similarly, a crosslinked PDMS has been generally obtained by mixing PDMSs containing vinyl or allyl and hydridosilyl groups, respectively, over the Pt catalyst, as well. The above similar reaction will occur when the polar molecule containing the vinyl or allyl groups is introduced into the PDMS mixture [24]. If the hydridosilyl group exists much more than the vinyl group in the mixture, the reaction between the polar molecule and the PDMS crosslinker would increase. As we control the mole ratio of vinyl to hydridosilyl groups in the mixture, we can increase permittivity as well as modulate degree of crosslinking of the film, resulting in improvement of its actuation strain. This is because the thickness strain for low strains (<20%) is proportional to the film permittivity and initial modulus according to the following equation [23,25] even if it does not fully explain large strain electro-mechanics due to strain nonlinearity [26,27,28]

*S*_z_= −ɛ_r_ɛ_0_*V*^2^/*E*

where *S*_z_ is the actuation strain or the thickness strain; ɛ_r_, the dielectric constant of the film; ɛ_0_, the permittivity of free space (8.854 pF/m); *V*, the electric field (volt per meter); and *E*, the initial modulus of the film.

In this work, we first control the mole ratio between vinyl and hydridosilyl groups in the PDMS mixture via copolymerization of each PDMS in order to chemically introduce a requisite molecule. It is preferable to the variation in wt % of PDMSs because the content of a PDMS crosslinker must preferentially affect mechanical properties of the PDMS elastomer. We synthesized high molecular weight poly(dimethylsiloxane-*co*-methylvinylsiloxane) (VPDMS) and poly(dimethylsiloxane-*co*-methylsiloxane) (HPDMS) from diethoxydimethylsilane and diethoxymethylvinylsilane or diethoxymethylsilane. We fixed the content of vinyl group in VPDMS and varied that of hydridosilyl group in HPDMS. We attempted a polar molecule containing vinyl or allyl groups to compete with VPDMS during crosslinking between VPDMS and HPDMS via the Pt-catalyzed hydrosilylation. We evaluated variations in permittivity, optical transparency, and initial modulus of the crosslinked film with the amount of hydridosilyl group in HPDMS and addition of tri(ethylene glycol) divinyl ether (TEGDE) as the polar chemical component into the mixture of VPDMS and HPDMS.

## 2. Materials and Methods

### 2.1. Materials

Diethoxydimethylsilane (**1**), diethoxymethylvinylsilane (**2**), and diethoxymethylsilane (**3**) were purchased from Tokyo Chemical Industry Co., Ltd. (Tokyo, Japan) Tri(ethylene glycol) divinyl ether (TEGDE), platinum (0)-1,3-divinyl-1,1,3,3,-tetramethyldisiloxane complex solution (0.05 mol % of platinum), and 37% hydrochloric solution in water were purchased from Sigma-Aldrich, Inc. (St. Louis, USA) Ethyl acetate (EA) and magnesium sulfate were purchased from Junsei Chemical Co., Ltd. (Tokyo, Japan). All reagents were used as received.

### 2.2. Synthesis of Poly(Dimethylsiloxane-co-methylvinylsiloxane) (VPDMS)

After 75.01 g of diethoxydimethylsilane and 0.82 g of diethoxymethylvinylsilane were introduced into 250 mL of 3-necked flask equipped with a stirrer under nitrogen, 7.9 mL of distilled water and 2.0 mL of 37% hydrochloric acid solution in water as a catalyst were dropwise added to the monomer mixture with stirring in a cold bath. The feeding mole ratio of the monomers was settled at 0.99:0.01 (**1**:**2**). The mixture was reacted at 70 °C with stirring of the rate of 250 rpm under a regulated nitrogen flow (70 mL/min) [29]. The polymerization was carried out until it is gelled. The highly viscous copolymer was dissolved in 200 mL of ethyl acetate (EA) and then the EA polymer solution was poured into 700 mL of water to remove the catalyst. The EA solution was separated from the excess water and dried by using magnesium sulfate. After the magnesium sulfate was filtered off and EA was removed by a vacuum evaporator at 35 °C, the transparent, colorless, and highly viscous polymer was dried at 35 °C under vacuum for 2 days. Yield: 33.8 g (89%). M_n_ = 10.1 × 10^4^ g/mol. PD = 1.64. l:m (mole ratio) = 0.990:0.010. IR ν_max_(Liquid, NaCl)/cm^−1^: 3055 w (=C–H str., vinyl); 2963 s, 2905 m (C–H str., methyl); 1598 w (C=C str., vinyl); 1445 w, 1412 m (C–H ben., methyl); 1261 s (Si–O–C str.) 1093 s, 1019 s (Si–O–Si str.). ^1^H NMR δ_h_ (CDCl_3_, 500 MHz): 5.92–6.05 (2H, m, vinyl); 5.78–5.83 (H, m, vinyl); 0.04–0.20 (9H, m, methyl). ^13^C NMR δ_c_ (CDCl_3_, 500 MHz): 137.11 (s, –CH=); 132.77 (s, =CH_2_); 0.76–1.35 (m, –CH_3_). IR, ^1^H, and ^13^C NMR spectra were shown in Figure S1 (in supplementary material).

### 2.3. Synthesis of Poly(Dimethylsiloxane-co-methylsiloxane) (HPDMS)

After diethoxydimethylsilane and diethoxymethylsilane were introduced into 250 mL of 3-necked flask equipped with a stirrer under nitrogen, 1.0 mol % of distilled water and 0.04 mol % of hydrochloric acid as a catalyst were dropwise added to the monomer mixture with stirring in a cold bath. The feeding mole ratios of the monomers were settled at 0.90:0.10 and 0.80:0.20 (**1**:**3**), respectively. The mixture was reacted at 70 °C with stirring of the rate of 250 rpm under a regulated nitrogen flow (70 mL/min). The polymerization was carried out until it is gelled. After that, the same procedure as above was performed.

HPDMS10 (**1**:**3** = 0.90:0.10, monomer feeding mole ratio). Diethoxydimethylsilane: 49.98 g. Diethoxymethylsilane: 5.02 g. H_2_O: 5.8 mL. 37% HCl solution in water: 1.5 mL. Yield: 23.8 g (87%). M_n_ =16.8 × 10^3^ g/mol. PD = 5.77. n:o (mole ratio)=0.925:0.075. IR ν_max_ (Liquid, NaCl)/cm^−1^: 2963 s, 2905 m (C–H str., methyl); 2156 m (Si–H str., hydridosilyl); 1445 w, 1412 m (C–H ben., methyl); 1261 s (Si–O–C str.); 1093 s, 1024 s (Si–O–Si str., siloxane). ^1^H NMR δ_h_ (CDCl_3_, 500 MHz): 4.71 (s, Si–H); 0.09–0.16 (m, –CH_3_). ^13^C NMR δ_c_ (CDCl_3_, 500 MHz): 0.62–1.37 (m, –CH_3_). IR, ^1^H, and ^13^C NMR spectra were shown in Figure S2 (in supplementary material).

HPDMS20 (**1**:**3** = 0.80:0.20, monomer feeding mole ratio). Diethoxydimethylsilane: 30.15 g. Diethoxymethylsilane: 6.82 g. H_2_O: 3.6 mL. 37% HCl solution in water: 1.0 mL. Yield: 16.01 g (64%). M_n_ = 16.4 × 10^3^ g/mol. PD = 2.34. n:o (mole ratio)=0.807:0.193. IR ν_max_ (Liquid, NaCl)/cm^−1^: 2964 s, 2905 m (C–H str., methyl); 2157s (Si–H str., hydridosilyl); 1446w, 1412m (C–H ben., methyl); 1261 s (Si–O–C str.); 1093s, 1027s (Si–O–Si str., siloxane). ^1^H NMR δ_h_ (CDCl_3_, 500 MHz): 4.70 (s, Si–H); 0.09–0.18 (m, Si–CH_3_). ^13^C NMR δ_c_ (CDCl_3_, 500 MHz): 0.76–1.33 (m, –CH_3_). IR, ^1^H, and ^13^C NMR spectra were shown in Figure S3.

### 2.4. Identification of Reaction of TEGDE with HPDMS

After ca. 0.0051 g of platinum (0)-1,3-divinyl-1,1,3,3,-tetramethyldisiloxane complex solution (0.05 mol % of platinum) was added to 0.5032 g of HPDMS10 and completely mixed, 0.1678 g of TEGDE was introduced into the mixture and thoroughly mingled together. The opaque dope layer was casted on a NaCl window. The layer was placed under vacuum at room temperature for 10 min to remove an air bubble in it and cured at 80 °C for 2 h under air. After that, it was placed under vacuum at 80 °C for 1 h to remove unreacted TEGDE. We compare IR spectra of the dope layer before and after curing.

### 2.5. Preparation of PDMS Dope and Film Fabrication

After ca. 0.25 wt % of platinum (0)-1,3-divinyl-1,1,3,3,-tetramethyldisiloxane complex solution (0.05 mol % of platinum) was added to VPDMS and completely mixed, HPDMS and TEGDE or HPDMS were introduced into the VPDMS mixture and thoroughly mingled together. The opaque PDMS dope was poured onto a glass plate and cast with a Doctor′s knife. The dope layer was placed under vacuum at room temperature for 10 min to remove an air bubble in the layer and cured at 80 °C for 2 h under air. After that, it was placed under vacuum at 80 °C for 1 h to remove unreacted TEGDE. We obtained a transparent film with a thickness of 140–150 µm.

### 2.6. Measurements

A Shimadzu UV-2600 spectrometer and a Nicolet 6700 FT-IR spectrometer were used for UV–vis and IR measurements, respectively. The liquid polymer or dope was coated on a NaCl window for the IR measurement. ^1^H and ^13^C NMR spectra were recorded with a Bruker 500 MHz NMR spectrometer. Chloroform-*d*_1_ was used as a solvent for the NMR measurements. The isothermal behavior of TEGDE was monitored using a TA Instruments TGA Q50 under nitrogen at 70, 80, and 90 °C, respectively. Mechanical properties of the films were measured by using TA Instrument RSA-G2. The films prepared from the polymer dopes were cut into a 5 × 30 mm strip. Initial length of the sample film was 10 mm. Five or six sample films were taken for each film and the samples with highest and lowest strain were excluded from the data analysis. The extension rate was 0.1 mm/s. Electrical properties of the film were obtained by using TA Instrument RSA-G2 and analyzed with an Agilent E4890A Precision LCR meter. The electrode was formed 20 mm in diameter on both sides of the film by vacuum-deposition of gold.

## 3. Results and Discussion

### 3.1. Synthesis and Characterization of VPDMS and HPDMS

**Scheme 1:**
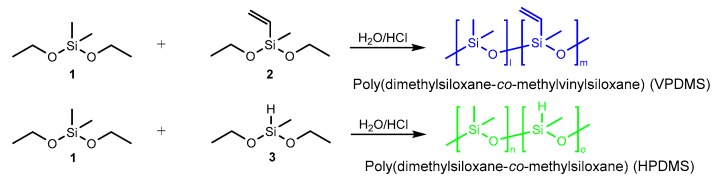
Synthesis of VPDMS and HPDMS.

We prepared two kinds of PDMS copolymer in order to form the crosslinked PDMS film via Pt-catalyzed hydrosilylation, poly(dimethylsiloxane-*co*-methylvinylsiloxane) (VPDMS), and poly(dimethylsiloxane-*co*-methylsiloxane) (HPDMS). VPDMS and HPDMS were synthesized as shown in Scheme 1. The methylvinylsiloxane moiety in VPDMS was settled at ca. 1 mol % because the amount seems to be enough for the film formation in comparison with a commercial product, Sylgard^TM^, which has the moiety only at the end in the main chain [30]. The methylsiloxane moiety in HPDMS was settled at ca. 10 and 20 mol %, respectively. The numbers appearing in each HPDMS sample name in Table 1 designate the feeding mol % of diethoxymethylsilane. Since HPDMS acts as a crosslinker, the amount of methylsiloxane moiety needs more than that of methylvinylsiloxane moiety in VPDMS in order to chemically introduce TEGDE in the crosslinking system. The copolymers were well identified in Figures S1–S3 (in supplementary material). As shown in Table 1, the copolymer composition was almost equal to the feeding mole ratio, which is calculated by ^1^H NMR spectroscopy. The calculation is based on the peak area near 0 ppm assigned to protons in Si–CH_3_ and 5.8–6.1 ppm assigned to protons in vinyl group for VPDMS, near 0 ppm assigned to protons in Si–CH_3_ and 4.7 ppm assigned to a proton in Si–H for HPDMS [14,31]. The detailed peak assignment of the ^1^H and ^13^C NMR spectra was summarized in the Materials and Methods section. HPDMS has much lower molecular weight than VPDMS does because it is gelled at an early polymerization stage. The copolymers were transparent, viscous liquids, as expected (Figure S4 in supplementary material).

### 3.2. Curing Condition of Crosslinking Between VPDMS and HPDMS

**Figure 1:**
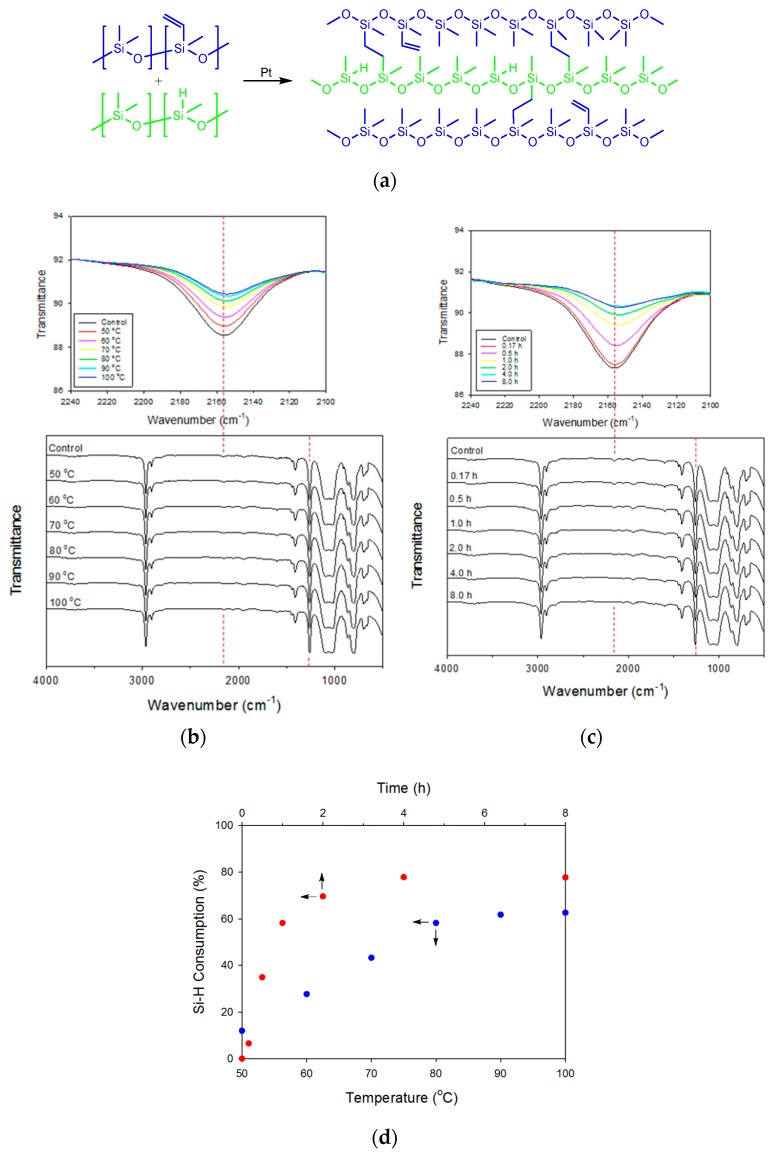
Schematically expressed crosslinking reaction between VPDMS and HPDMS (**a**). IR spectrum variation of a VH10 dope layer with curing temperature (**b**) and time (**c**). Degree of crosslinking calculated from the IR spectra (**d**).

We prepared three kinds of the crosslinked film by using the polymer dopes as shown Table 2. Composition of the dopes was settled at 9:1:1 (wt %) between, VPDMS, TEGDE, and HPDMS, respectively. In the case of VH10 prepared only from VPDMS and HPDMS, the vinyl group in VPDMS and the hydridosilyl group in HPDMS are reacted each other in the presence of platinum (Pt) as a catalyst, resulting in crosslinking reaction between two polymers as shown in Figure 1a. This reaction is well-known as Pt-catalyzed hydrosilylation. The hydrosilylation reaction has been used as a method of modifying silicone elastomers [31,32,33,34,35].

First of all, we tried to examine a proper curing condition for our material system. Figure 1b,c show variations in IR spectrum of the dope layer with curing temperature and time, respectively. The VH10 dope was coated on a NaCl window and cured at each temperature for 1 h. After that, we fixed the curing temperature, 80 °C, and cured for each time. The absorption peak at near 2160 cm^−1^ is due to Si–H stretching vibration of HPDMS [29,31,34]. This peak intensity decreases as the curing temperature or time increases because the hydridosilyl group is consumed as the crosslinking reaction occurs. The Si–H consumption, which relates to the degree of crosslinking, was calculated from the peak variation. The intensity evolution of the Si–H group can be determined from the ratio change of absorption peak intensity between 2160 and 1260 cm^−1^ over temperature or time. The absorption peak near 1260 cm^−1^ is assigned to Si–CH_3_ stretching vibration of VPDMS and HPDMS. The quantitative analysis of the Si-H consumption is based on this peak intensity because the concentration of Si–CH_3_ group is not changed during crosslinking [36,37]. The consumption is evolved as shown in Figure 1d. It was almost maximized at 80 °C, ca. 58%, when the dope layer was cured for 1 h. It was no more than 63% at 100 °C. At 80 °C, two hours of the curing time were enough for obtaining high degree of crosslinking, as well. The Si-H consumption showed ca. 70% when the layer was cured at 80 °C for 2 h. In this case, the amount of vinyl group is slightly higher than that of the hydridosilyl group in the dope because the mole ratio between them is calculated at 1.14 (vinyl/ hydridosilyl groups) (Table 2).

### 3.3. Fabtication of PDMS Elastomer Films Containing TEGDE

**Figure 2:**
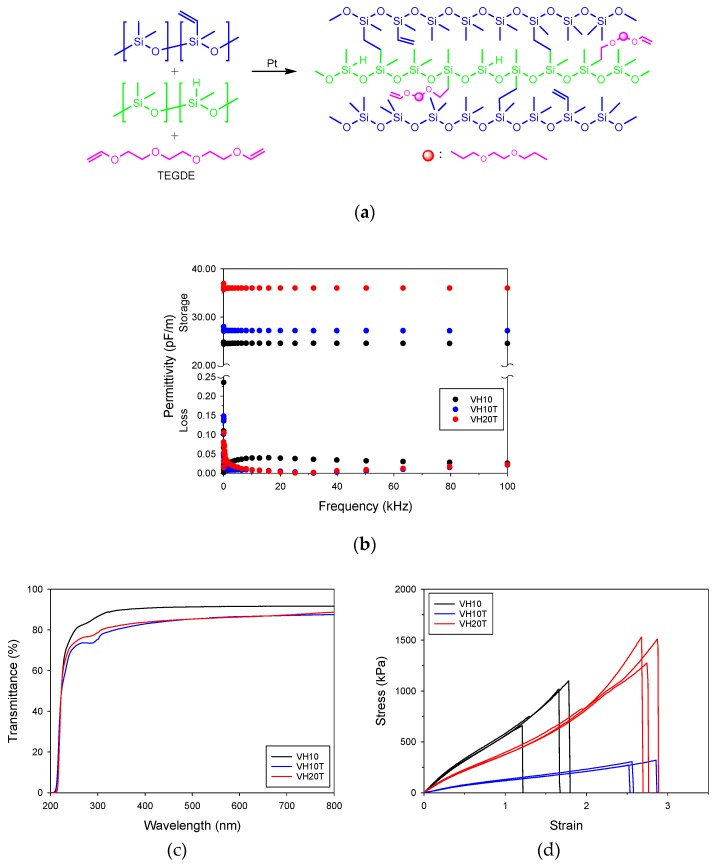
Schematically expressed reaction of TEGDE with HPDMS during crosslinking between VPDMS and HPDMS (**a**). Storage permittivity (**b**), transmittance (**c**), and stress–strain curves (**d**) of the crosslinked films fabricated from VH10, VH10T, and VH20T polymer dopes, respectively.

The polymer dope layer containing TEGDE was also cured at 80 °C for 2 h according to the above results (Figure 1d) we obtained. We have known from the result in Figure S5 (in supplementary material) that a vinyl group of TEGDE is also reacted to the hydridosilyl group in HPDMS in the presence of Pt. After curing at 80 °C for 2 h, the hydridosilyl group in HPDMS was consumed at ca. 63%. The mole ratio of the vinyl to hydridosilyl groups was calculated at 1 to 0.311. TEGDE can be slowly vaporized at high temperature. The vaporization rate of TEGDE was measured at ca 0.1 mg/min at 80 °C as shown in Figure S6 (in supplementary material). Therefore TEGDE would exist enough for reacting to HPDMS in the dope layer of VH10T or VH20T all the curing time. Figure 2a shows a probable chemical reaction when TEGDE is added to the mixture prepared from VPDMS and HPDMS. In this case, therefore, the vinyl group of TEGDE can be competed with that of VPDMS to react to the hydridosilyl group in HPDMS. TEGDE has two vinyl groups at the end, resulting in increasing the possibility of reacting with HPDMS. Figure S7 (in supplementary material) shows the absorption peak near 2160 cm^−1^ of not only the VH10T but also the VH20T dope layers completely disappears due to a chemical participation of TEGDE into HPDMS during curing even though the peak of the VH10 dope layer remains after curing. We can see that the IR spectra and the mole ratio between vinyl and hydridosilyl groups of VH10T and VH20T dopes in Table 2 that TEGDE was more reacted in the VH20T than in the VH10T. 

### 3.4. Electrical, Optical, and Mechanical Properties of the PDMS Elastomer Films

Figure 2b shows that storage permittivity of a crosslinked PDMS film can be increased by chemical introduction of a polar moiety into HPDMS. In our case, storage permittivity of the crosslinked PDMS film is readily increased from ca. 25 to 36 pF/m at 10 kHz. This property will increases more as the amount of hydridosilyl group increases in HPDMS. It is well known from previous reports [15,19,20,23] that the storage permittivity of PDMS is ca. 23–26 pF/m at 10 kHz. Loss permittivity of the crosslinked PDMS film slightly fluctuated within 0.01–0.04 pF/m at 1–100 kHz. We obtained transparent films from the dope layers of VH10, VH10T, and VH20T, respectively, as shown in Figure 2c. Transmittance of the crosslinked film obtained only from VPDMS and HPDMS was ca. 93% in a visible region. The crosslinked PDMS film containing TEGDE showed almost 90% of transmittance even though the dope layer containing TEGDE was opaque due to the big difference of polarity between PDMS and TEGDE. This is because TEGDE is chemically introduced into the crosslinked PDMS film during curing and unreacted TEGDE is completely removed under vacuum.

The degree of crosslinking of PDMS film would decrease if VPDMS compete against TEGDE in reacting to HPDMS. Therefore, the initial modulus (*E*) and the strain (ɛ) of the crosslinked VH10T film is decreased from 760 to 150 MPa and increased from 160% to 270%, respectively, as TEGDE is added to the VH10 dope layer, as shown in Figure 2d. Since the crosslinking reaction between VPDMS and HPDMS as well as the TEGDE chemical participation into the film would be facilitated by increase of the hydridosilyl group in the HPDMS main chain, the initial modulus of the crosslinked VH20T film becomes higher than that of the VH10T film. The mechanical properties of the crosslinked PDMS films were summarized in Table 2. We expect from the result that the modified PDMS films are superior to the commercial ones such as Sylgard^TM^ and VHB^TM^ in terms of the actuation efficiency because Sylgard^TM^ and VHB^TM^ films are much harder and softer than the films, respectively [14,38]. The actuation strain of the VHB^TM^ film would be higher than that of the crosslinked PDMS films because it has several tens of kPa of initial modulus. However, since VHB^TM^ film readily yields [14], the film can be wrinkled or creased during repeated actuation.

## 4. Conclusions

We developed a facile, effective method for functionalizing a PDMS elastomer film. We simply chemically introduced a molecule desirous of acquiring a special property into the PDMS film during crosslinking reaction between poly(dimethylsiloxane-*co*-methylvinylsiloxane) (VPDMS) and poly(dimethylsiloxane-*co*-methylsiloxane) (HPDMS) via Pt-catalyzed hydrosilylation. The functionalization of the crosslinked PDMS film comes from the competition reaction between vinyl groups in VPDMS and in the additive to a hydridosilyl group in HPDMS. We demonstrated that the permittivity and mechanical properties of the crosslinked PDMS film were significantly improved to be suitable for an electrical-driven actuator by chemically introducing tri(ethylene glycol) divinyl ether (TEGDE) as a polar molecule. In addition, the film shows high transparency due to the chemically introduction of TEGDE into it even though TEGDE and the liquid PDMS mixture are immiscible. First of all, these improvements can be conveniently achieved by adding the requisite chemical component into the mixture of VPDMS and HPDMS. Future work will focus on investigating the most efficient ratio between the crosslinking groups in VPDMS and in HPDMS in consideration of the competition reaction with an additional molecule.

## Figures and Tables

**Table 1:** Characterization of VPMDS and HPDMS

Name of Copolymer	M_n_ (g/mol) ^1^	M_w_ (g/mol) ^1^	l or n (Mole Ratio)	m or o (Mole Ratio)
VPDMS	100,741	165,425	0.990	0.010
HPDMS10	16,814	97,048	0.925	0.075
HPDMS20	16,414	38,480	0.807	0.193

^1^ M_n_ and M_w_ mean the number- and the weight-average molecular weight, respectively.

**Table 2:** Composition of polymer dopes and mechanical properties of the crosslinked PDMS films cured at 80 °C for 2 h

Name of Polymer Dope	VPDMS (g)	TEGDE (g)	HPDMS10 (g)	HPDMS20 (g)	Initial Modulus ^1^ (kPa) (s.d.) ^2^	Maximum Stress ^1^ (kPa) (s.d.)	Maximum Strain ^1^ (%) (s.d.)
VH10	1.3769	-	0.1589 (1.14) ^3^	-	762 (54)	926 (234)	156 (30)
VH10T	1.4542	0.1685	0.1697 (1.12) ^3^	-	154 (17)	299 (25)	265 (18)
VH20T	1.3554	0.1547	-	0.1509 (0.448) ^3^	484 (36)	1,438 (141)	277 (10)

^1^ The values were obtained from Figure 2d. ^2^ s.d. means standard deviation. ^3^ The mole ratio between vinyl in VPDMS and hydridosilyl groups in HPDMS (vinyl/ hydridosilyl groups).

## Supplementary Materials

The following are available online at https://www.mdpi.com/2073-4360/11/11/1842/s1, Figure S1: IR (a), ^1^H NMR (b), ^12^C NMR (c), and GPC spectra (d) of VPDMS; Figure S2: IR (a), ^1^H NMR (b), ^12^C NMR (c), and GPC spectra (d) of HPDMS10; Figure S3: IR (a), ^1^H NMR (b), ^12^C NMR (c), and GPC spectra (d) of HPDMS20; Figure S4: A photograph of the synthesized PDMS copolymers; Figure S5: IR spectrum of the dope layer prepared from HPDMS10 and TEGDE before and after curing at 80 °C for 2 h; Figure S6: Isothermal TGA curves of TEGDE at different temperatures; Figure S7: IR spectra of the dope layers prepared from VH10 (a), VH10T (b), VH20T (c) before and after curing at 80 °C for 2 h.
