# Supplementary material for: Facile Functionalization of Poly(Dimethylsiloxane) Elastomer by Varying Content of Hydridosilyl Groups in a Crosslinker
Source: Polymers (Basel). 2019 Nov 8;11(11):1842. doi: 10.3390/polym11111842 (PMC6918333; doi:10.3390/polym11111842)
Supplement: Supplementary file 1 [file polymers-11-01842-s001.pdf]

Supplementary Materials

Facile Functionalization of Poly(dimethylsiloxane) Elastomer by Varying Content of Hydridosilyl Groups in a Crosslinker

Seung Koo Park [1,*], Bong Je Park [1], Mee Jeong Choi [1], Dong Wook Kim [2], Jae Woong Yoon [1], Eun Jin Shin [1], Sungryul Yun [1], and Suntak Park [1,*]

[1] Human Enhancement & Assistive Technology Research Section, Artificial Intelligence Research Laboratory, Electronics and Telecommunications Research Institute, 218 Gajeong-ro, Yuseong-gu, Daejeon 34129, South Korea; bjpark@etri.re.kr (B.J.P.); jjeong0527@etri.re.kr (M. J. C.); yjw60212@etri.re.kr (J.W.Y.); shin015511@etri.re.kr (E.J.S.); sungryul@etri.re.kr (S.Y.)

[2] Advanced Materials Division, Korea Research Institute of Chemical Technology, 141 Gajeong-ro, Yuseong-gu, Daejeon 34114, South Korea; dongwook@krict.re.kr

* Correspondence: skpark@etri.re.kr (S.K.P.); spark@etri.re.kr (S.P.)

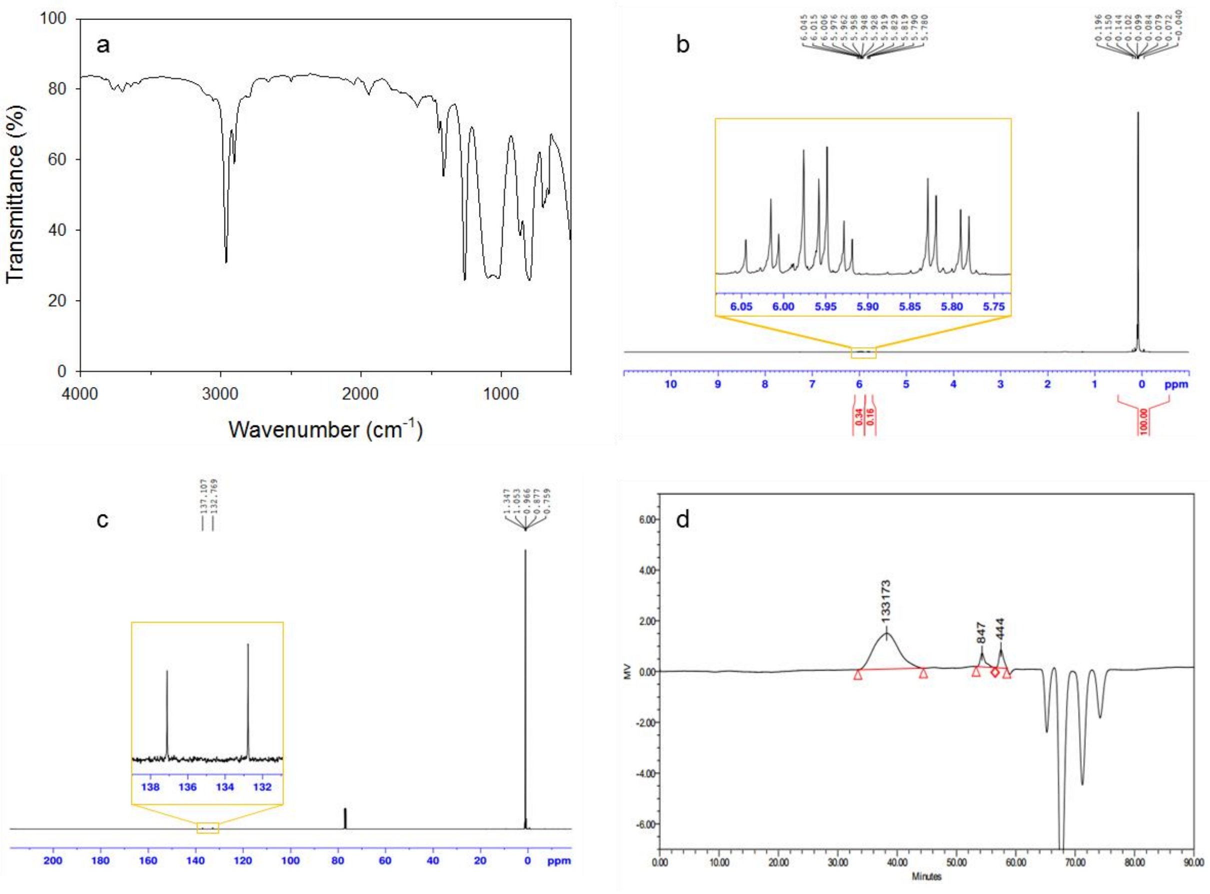

Figure S1. IR (a), ^{1}H NMR (b), ^{12}C NMR (c), and GPC spectra (d) of VPDMS.

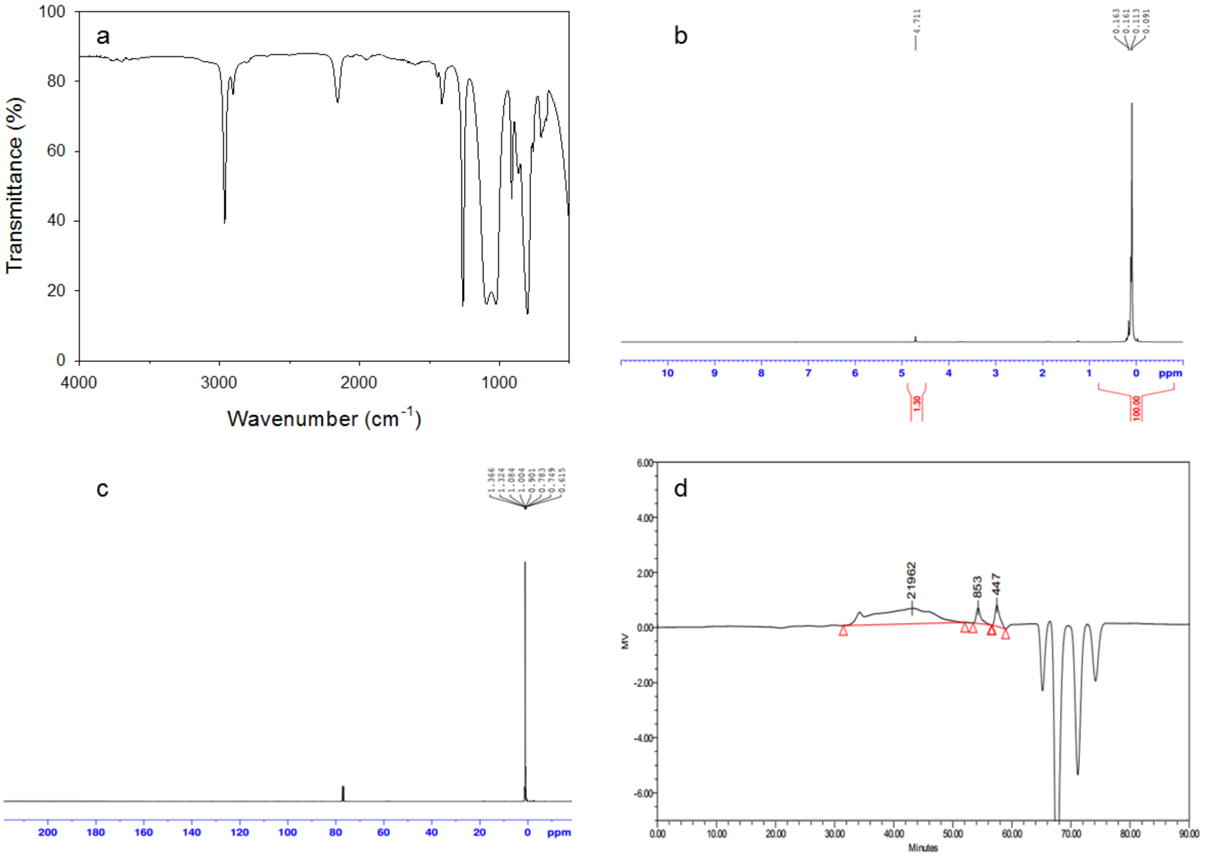

Figure S2. IR (a), ^{1}H NMR (b), ^{12}C NMR (c), and GPC spectra (d) of HPDMS10.

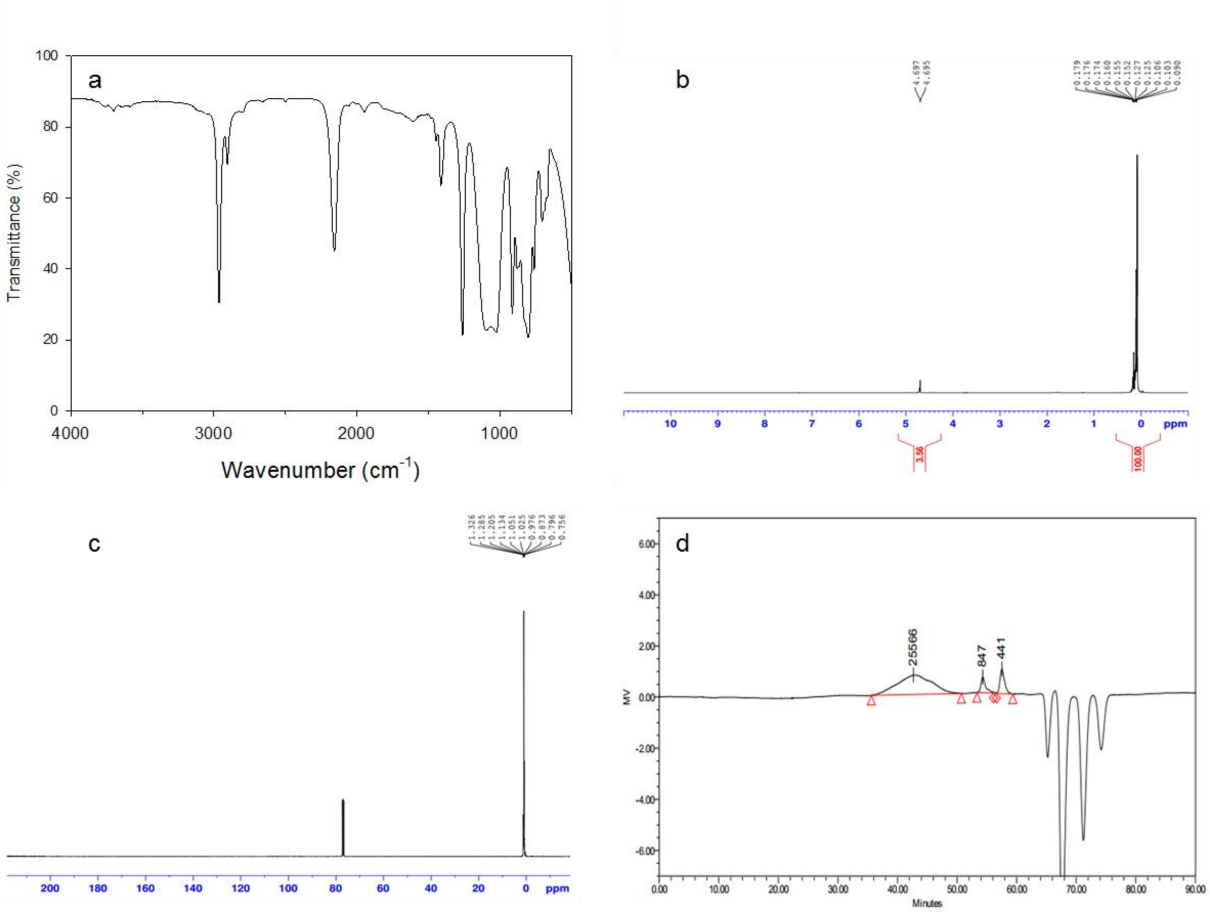

Figure S3. IR (a), ^{1}H NMR (b), ^{12}C NMR (c), and GPC spectra (d) of HPDMS20.

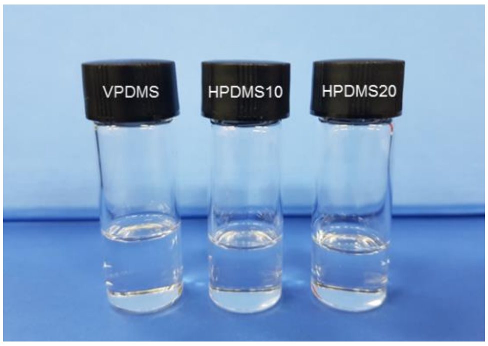

Figure S4. A photograph of the synthesized PDMS copolymers.

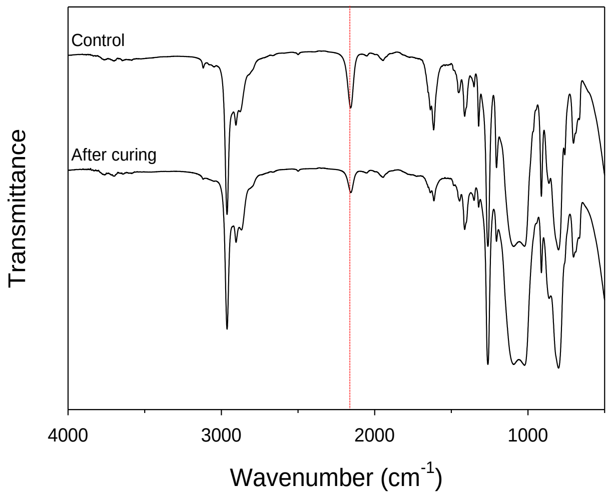

Figure S5. IR spectrum of the dope layer prepared from HPDMS10 and TEGDE before and after curing at 80 ℃ for 2h.

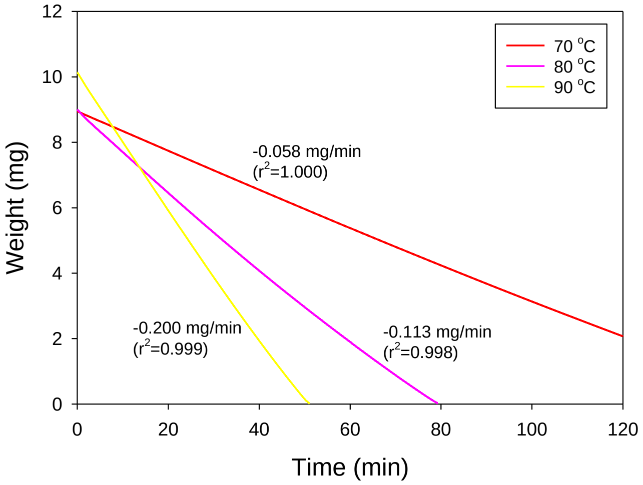

Figure S6. Isothermal TGA curves of TEGDE at different temperatures.

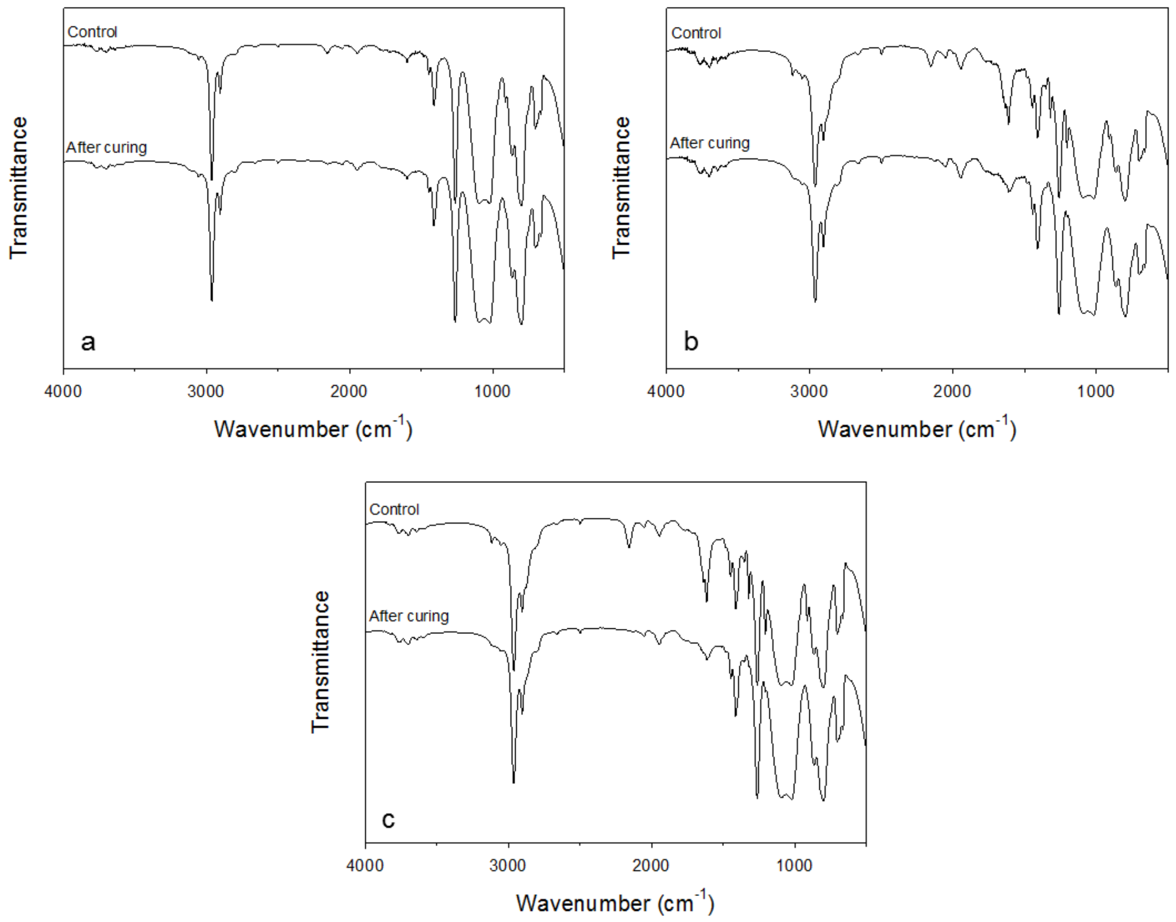

Figure S7. IR spectra of the dope layers prepared from VH10 (a), VH10T (b), VH20T (c) before and after curing at 80 ℃ for 2 h.
